# Aldosterone: From Essential Tubular Regulator to Pathological Driver—Physiology, Disease, and Therapeutic Advances

**DOI:** 10.3390/ijms26188829

**Published:** 2025-09-10

**Authors:** Camillo Tancredi Strizzi, Viola D’Ambrosio, Giuseppe Grandaliano, Francesco Pesce

**Affiliations:** 1 Department of Translational Medicine and Surgery, Università Cattolica del Sacro Cuore, 00168 Rome, Italy; camillotancredi.strizzi01@icatt.it (C.T.S.); viola.dambrosio@policlinicogemelli.it (V.D.); giuseppe.grandaliano@policlinicogemelli.it (G.G.); 2 Nephrology, Dialysis and Transplantation Unit, Fondazione Policlinico Universitario A. Gemelli IRCCS, 00168 Rome, Italy; 3 Division of Renal Medicine, Ospedale Isola Tiberina—Gemelli Isola, 00186 Rome, Italy

**Keywords:** aldosterone, mineralocorticoid receptor, aldosterone-sensitive distal nephron, aldosterone breakthrough, chronic kidney disease, precision nephrology

## Abstract

Aldosterone is a key regulator of sodium reabsorption, potassium secretion, and acid–base balance along the aldosterone-sensitive distal nephron (ASDN), where it exerts coordinated, segment-specific control over tubular transport. Although essential for volume conservation in terrestrial environments, aldosterone signaling has become maladaptive in modern sodium-rich contexts, contributing to systemic inflammation, fibrosis, and progression of cardiovascular and kidney disease. This review examines the regulation of aldosterone biosynthesis, the molecular diversity of mineralocorticoid receptor (MR) signaling, and the cellular mechanisms by which aldosterone shapes ion transport in the ASDN. A detailed classification of aldosterone-related disorders is presented, including hyperaldosteronism, pseudo-hyperaldosteronism, aldosterone resistance, and hypoaldosteronism. The therapeutic section focuses on MR overactivation in chronic kidney disease, critically appraising the clinical use of steroidal and non-steroidal MR antagonists. In addition, emerging strategies targeting aldosterone synthesis and downstream inflammatory pathways are discussed as potential approaches to address residual cardiorenal risk and the aldosterone breakthrough phenomenon. Together, these insights support a mechanistic reappraisal of aldosterone as both a physiological modulator and a pathologic driver, with implications for biomarker-guided, targeted therapy.

## 1. Introduction

**Figure 1:**
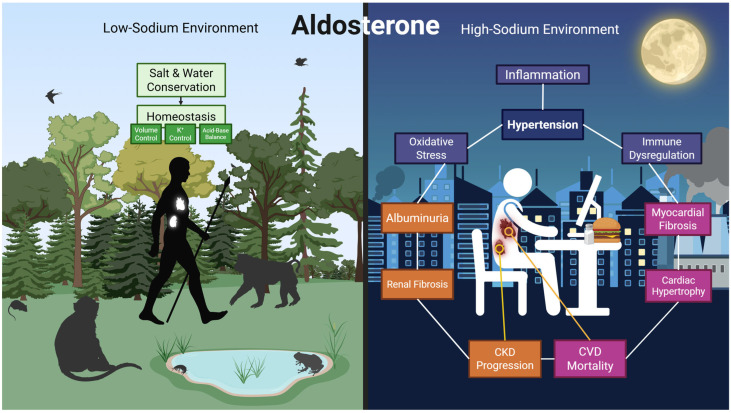
**The dual role of aldosterone.** The role of aldosterone is defined by the environment. In ancestral, low-sodium settings, aldosterone was essential for survival, maintaining homeostasis by promoting salt and water conservation, potassium (K^+^) control, and acid–base balance. In the context of modern high-sodium diets, this once-beneficial signaling has become maladaptive. It now drives systemic inflammation, fibrosis, and oxidative stress, contributing to hypertension, myocardial damage, albuminuria, progression of chronic kidney disease (CKD), and overall cardiovascular (CVD) mortality.

In the complex physiology of vertebrate life, the maintenance of fluid and electrolyte homeostasis is fundamental for survival [1]. At the core of this regulatory network is aldosterone, a mineralocorticoid steroid hormone that was first identified in the 1950s [2]. Aldosterone exerts its principal effects by promoting sodium and water reabsorption and facilitating the excretion of potassium and hydrogen ions, regulating blood pressure, extracellular fluid volume, and acid–base equilibrium [3,4]. The evolutionary emergence of aldosterone can be traced back to a gene duplication event that occurred approximately 420 million years ago in aquatic vertebrates [5]. In these organisms, the gene played a role in response to hypoxic stress [5]. The evolutionary adaptation of aldosterone for the conservation of salt and water on land was instrumental in the ecological success of tetrapods, enabling them to thrive and diversify across environments that became increasingly osmotically demanding [5,6]. However, in the modern era, the physiological role of aldosterone has become more problematic. A system originally shaped to preserve salt and water in environments where sodium was scarce has become maladaptive in the context of today’s sodium-rich diets [7]. As a result, aldosterone levels that fall within the normal range are increasingly associated with the development of hypertension and its complications, including left ventricular hypertrophy, myocardial fibrosis, heart failure with preserved ejection fraction (HFpEF), chronic kidney disease, and increased cardiovascular mortality [7,8,9] (Figure 1).

This review will explore the multifaceted nature of aldosterone, bridging its fundamental physiology with its complex role in clinical disease. We will first examine the intricate molecular mechanisms governing its synthesis and the diverse signaling pathways it activates. Subsequently, we will detail the clinical syndromes arising from both aldosterone excess and deficiency. Finally, we will focus on established and emerging therapeutic interventions designed to counter the hormone’s detrimental effects, providing a comprehensive overview from cellular function to modern therapeutic strategies.

## 2. The Pathophysiology of Aldosterone

### 2.1. Regulation of Aldosterone Synthesis

**Figure 2:**
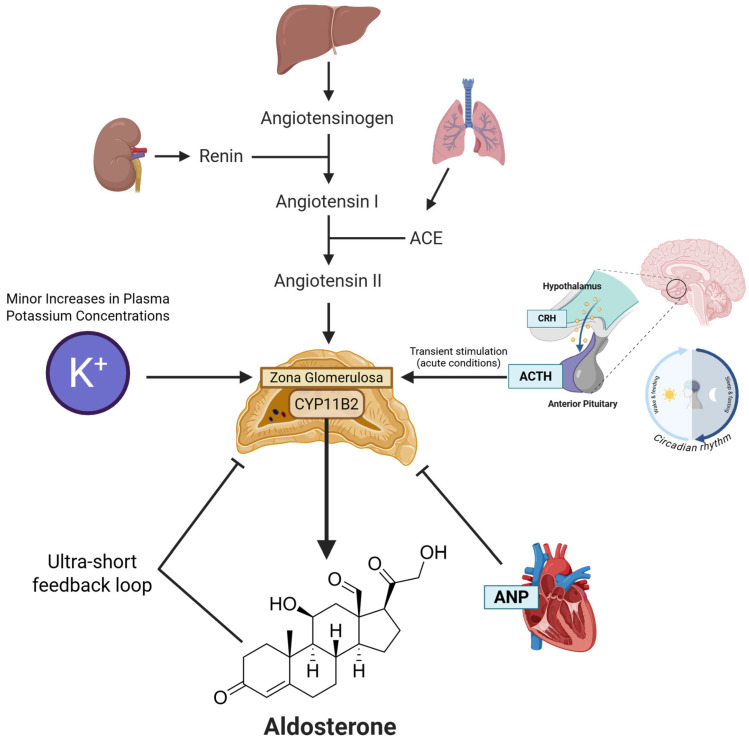
**Regulation of aldosterone biosynthesis.** Aldosterone synthesis, catalyzed by aldosterone synthase (CYP11B2) in the adrenal zona glomerulosa, is tightly controlled. The process is primarily stimulated by the renin–angiotensin system, which produces angiotensin II to act on the adrenal gland. Direct stimulation also occurs in response to small increases in plasma potassium (K^+^). Adrenocorticotropic hormone (ACTH) mediates transient, stress-related release and is responsible for the hormone’s circadian rhythm. Key inhibitory signals include atrial natriuretic peptide (ANP), which is released by the heart to counter volume expansion, and a local negative feedback loop from aldosterone itself. Abbreviations: ACE, angiotensin-converting enzyme; ACTH, adrenocorticotropic hormone; ANP, atrial natriuretic peptide; CRH, corticotropin-releasing hormone.

Aldosterone biosynthesis is confined to the zona glomerulosa of the adrenal cortex, where its production is determined by the expression of the gene CYP11B2 which encodes for aldosterone synthase [10]. Unlike cortisol synthesis, which is predominantly under hypothalamic–pituitary–adrenal axis control, aldosterone regulation is driven primarily by the renin–angiotensin–aldosterone system (RAAS) and extracellular potassium concentrations [11]. The convergence of these regulatory inputs is summarized in Figure 2.

The RAAS cascade is initiated by the release of renin from the granular cells of the renal juxtaglomerular apparatus. Renin secretion is stimulated by three main canonical pathways: decreased NaCl delivery to the macula densa, via nitric oxide and COX-2-dependent pathways; reduced perfusion pressure in the afferent arteriole; and direct sympathetic stimulation via β_1_-adrenergic receptors [12]. The RAAS cascade culminates in angiotensin II, which binds to AT1 receptors on zona glomerulosa cells. This activates the phospholipase C pathway, increasing intracellular calcium to potently stimulate CYP11B2 transcription and aldosterone synthesis [13,14]. AT1 activation also drives vasoconstriction and renal sodium retention, amplifying blood pressure restoration; in contrast, AT2 receptors counterbalance this response through vasodilation and anti-proliferative effects [15]. Of note, alternatively, non-ACE pathways involving enzymes such as chymase, cathepsin D, and tissue plasminogen activator (tPA) can also generate angiotensin peptides, providing potential for tissue-specific RAAS modulation [15].

Independently of the RAAS, aldosterone secretion is powerfully and directly stimulated by minor increases in plasma potassium (K^+^) concentration [16]. An elevation in extracellular K^+^ depolarizes the zona glomerulosa cell membrane, which activates voltage-gated Ca^2+^ channels. The resulting influx of extracellular Ca^2+^ is a potent stimulus for CYP11B2 gene expression and the enzymatic conversion of cholesterol to aldosterone [17]. This direct K^+^-sensing mechanism allows for rapid adjustments in aldosterone secretion to facilitate renal potassium excretion without necessarily engaging the entire RAAS cascade.

Furthermore, adrenocorticotropic hormone (ACTH) has been observed to transiently stimulate aldosterone secretion in the zona glomerulosa, particularly under conditions of acute stress, via a cyclic AMP (cAMP)-dependent pathway [18,19]. This mechanism is also crucial for maintaining the circadian rhythm of aldosterone secretion, which largely parallels that of cortisol. Studies employing dexamethasone to suppress endogenous ACTH have demonstrated that this rhythm is consequently abolished, thus confirming ACTH’s dominant role over the RAAS in generating this daily pattern [20]. In contrast, prolonged exposure to ACTH appears to favor glucocorticoid over mineralocorticoid production [21]. It is worth noting that in certain forms of primary aldosteronism, particularly in aldosterone-producing adenomas (APA), aldosterone secretion can become abnormally dependent on the diurnal rhythm of endogenous ACTH [22]. Recent molecular studies have proven that APAs with ATPase variants (ATP1A1/ATP2B3) are highly responsive to ACTH, a trait linked to robust expression of the ACTH receptor (MC2R) and its accessory protein, MRAP. Conversely, APAs with the common KCNJ5 variant express lower levels of MC2R, likely explaining their more modest response to ACTH stimulation [23].

Recent evidence has also identified the zona glomerulosa-derived protein Klotho as an important local modulator of aldosterone synthesis. Specifically, targeted Klotho depletion within zona glomerulosa cells enhances CYP11B2 expression by upregulating the transcription factors NGFIB and Nurr1 [19].

Inhibitory signals also provide crucial regulatory input. Atrial natriuretic peptide (ANP), released from cardiac atria in response to mechanical stretch caused by volume expansion, directly suppresses aldosterone secretion, thereby promoting natriuresis and contributing to volume homeostasis [24,25]. Furthermore, evidence suggests the existence of an ultra-short negative feedback loop, whereby aldosterone can act on local mineralocorticoid receptors (MR) within the adrenal gland to inhibit its own production, adding another layer of fine-tuning to the system [26]. Moreover, transcription factor Early Growth Response 1 (EGR1) was recently identified as a negative modulator of CYP11B2 expression in adrenal cells, under conditions of oxidative stress [27].

Recent advances have highlighted the crucial role of epigenetic regulation in controlling aldosterone synthesis, particularly through dynamic DNA methylation changes at the CYP11B2 promoter [28]. The methylation status of specific CpG dinucleotides inversely correlates with CYP11B2 expression, effectively acting as a molecular brake [29]. Under physiological stimuli, such as a low-salt diet, potassium elevation, or Ang II exposure, active demethylation occurs, increasing transcriptional accessibility and responsiveness of the CYP11B2 promoter [30].

### 2.2. The Mineralocorticoid Receptor

A central tenet of mineralocorticoid physiology is the mechanism that confers aldosterone selectivity upon the MR. The MR itself exhibits an equally high affinity (~0.5 nM) for aldosterone and for the glucocorticoid hormone cortisol, which circulates at concentrations 100- to 1000-fold higher in the plasma [31]. This issue is resolved by the tissue-specific expression of the enzyme 11β-hydroxysteroid dehydrogenase type 2 (11β-HSD2), which functions as a highly efficient gatekeeper, catalyzing the rapid, NAD^+^-dependent conversion of cortisol to its non-MR-affine 11-keto metabolite, cortisone [32]. In tissues that express MR but have low or negligible 11β-HSD2 activity, such as the heart, brain, and vasculature, and in cell types including cardiomyocytes, vascular smooth muscle cells, and macrophages, the MR is largely unprotected [31,33]. The MR can be activated by physiological or stress-induced elevations in cortisol [34]. This cortisol-mediated MR activation is now recognized as a key driver of cardiovascular and renal pathology [34]. What was once considered “aldosterone-driven” cardiac or vascular damage is now understood to be “MR-driven” pathology, which can be activated by either aldosterone or, in the absence of 11β-HSD2, the far more abundant cortisol [31,35]. In the heart, for instance, MR activation in cardiomyocytes and cardiac fibroblasts promotes myocardial fibrosis, leading to diastolic dysfunction and contributing to the pathophysiology of HFpEF [36,37,38]. Within the vasculature, MR signaling in endothelial and vascular smooth muscle cells enhances oxidative stress and reduces nitric oxide bioavailability, promoting endothelial dysfunction, a key initiating event in atherosclerosis [39]. Furthermore, MR expression in immune cells, particularly macrophages, is a critical driver of systemic inflammation; its activation leads to the recruitment of inflammatory cells into target tissues like the heart and blood vessels, amplifying tissue damage and adverse remodeling [35,40]. This mechanistic insight underscores the therapeutic rationale for MR antagonists (MRAs) in treating cardiovascular diseases, as they block the deleterious effects of receptor activation regardless of the activating ligand [36,41].

### 2.3. Genomic and Non-Genomic Pathways

The classical mechanism of aldosterone action is initiated by its passive diffusion across the plasma membrane of target epithelial cells. In the cytoplasm, aldosterone binds to the MR, which is held in an inactive, high-affinity conformation by a multiprotein chaperone complex that includes heat shock protein 90 (HSP90), HSP70, and various immunophilins such as FKBP51 and FKBP52 [42,43]. When translocated to the nucleus, the resulting “aldosterone-induced proteins” can be categorized into early- and late-response gene products. Among the most critical early-response genes is the serum and glucocorticoid-regulated kinase 1 (SGK1) [44]. The SGK1 mRNA is detectable within 30 min of aldosterone exposure, and the translated protein acts as a pivotal kinase [45]. SGK1 phosphorylates and inactivates the E3 ubiquitin ligase NEDD4-2 [46]. By inhibiting NEDD4-2, aldosterone prevents the ubiquitination and subsequent endocytosis and degradation of the epithelial sodium channel (ENaC), thereby increasing the channel’s half-life and density at the apical membrane and promoting sustained sodium reabsorption [44]. The late phase of the genomic response involves the de novo synthesis of the protein subunits that constitute ENaC (α, β, and γ) and the Na^+^/K^+^-ATPase [47,48]. This further augments the cell’s capacity for vectorial sodium transport. Due to the requirements for transcription and translation, this genomic pathway has a characteristic latency period, with physiological effects on ion transport becoming apparent 30–90 min after hormone exposure and lasting for several hours [49,50]. Recent evidence expands SGK1′s physiological role beyond ENaC regulation, highlighting its involvement in endothelial actin polymerization and increased vascular stiffness, particularly under high-salt conditions [51]. Moreover, SGK1 modulates non-selective cation channels such as TRPM7 and TRPV4, suggesting a broader regulatory function affecting distal tubule calcium and magnesium homeostasis [45].

Increasing evidence indicates that aldosterone can also elicit rapid physiological responses (occurring within seconds to minutes) that are incompatible with the timeline of gene transcription [52,53]. These non-genomic effects are mediated by the activation of a sub-population of MR located at the plasma membrane or within the cytoplasm, which engage classic second messenger signaling cascades [54]. In addition, aldosterone has been shown to rapidly activate pathways involving protein kinase C (PKC), mitogen-activated protein kinases (MAPK) such as ERK1/2, and the generation of inositol 1,4,5-trisphosphate (IP_3_) [55,56,57,58]. Recent evidence highlights that a significant proportion of aldosterone’s acute effects involves alternative membrane-associated receptors distinct from classical MR, such as the G protein-coupled estrogen receptor (GPER/GPR30) [59,60]. Additionally, aldosterone rapidly induces interactions between membrane MR and angiotensin II type 1 receptor (AT1R), engaging kinases like GRK5 and promoting immediate cellular hypertrophic responses independently of transcription [61].

**Figure 3:**
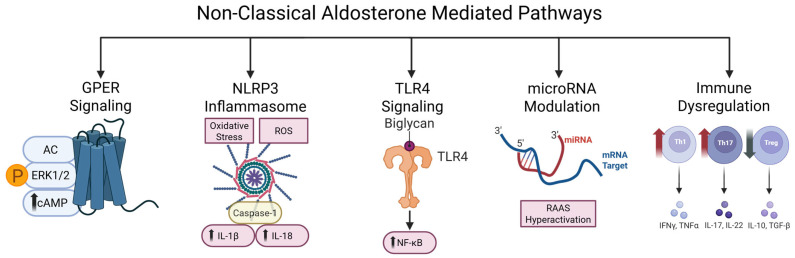
**Non-classical aldosterone-mediated pathways**. (1) Activation of the G protein-coupled estrogen receptor (GPER), leading to ERK1/2 phosphorylation and cAMP signaling; (2) blockade of NLRP3 inflammasome activation, triggered by oxidative stress and reactive oxygen species (ROS), which drives caspase-1-dependent release of IL-1β and IL-18; (3) inhibition of Toll-like receptor 4 (TLR4) signaling activated by extracellular biglycan, which promotes NF-κB–mediated inflammation; (4) modulation of microRNAs (miRNAs), either through mimics or antagomirs, to correct RAAS hyperactivation at the post-transcriptional level; and (5) immunomodulatory approaches that counter aldosterone-induced immune imbalance by downregulating Th1/Th17 cells and enhancing regulatory T cell (Treg) responses.

Aldosterone-driven non-classical downstream effects (Figure 3) include the promotion of inflammation through direct activation of immune cells such as macrophages and T lymphocytes, promoting the expression of cytokines (TNF-α, IL-6, IL-1β) and adhesion molecules (ICAM-1, VCAM-1) while also enhancing oxidative stress via NADPH oxidase activation [7,62,63], and NLRP3 inflammasome priming [64,65,66]. Experimental work has shown that aldosterone activates the NF-κB signaling pathway in the renal collecting duct, leading to increased expression of multiple pro-inflammatory and pro-fibrotic genes, including IL-1β, IL-6, and plasminogen activator inhibitor-1 (PAI-1) [67]. The upregulation of cytokines and adhesion molecules enables monocytes to infiltrate the subendothelial space, where they differentiate into macrophages and contribute to the formation of atherosclerotic plaques [39]. In mouse models in which the MR in macrophages is incapable of DNA binding, cardiac tissue inflammation still develops in response to a deoxycorticosterone/salt challenge, indicating that the early stages of cardiac inflammation can be driven by non-DNA-binding MR actions alone [68]. Experimental evidence has shown that selective deletion of MR in myeloid cells limits the accumulation of macrophages and vascular inflammation, thereby inhibiting neointimal hyperplasia and vascular remodeling [69]. In addition to macrophages, experimental studies have shown that dendritic cells express MR. In response to aldosterone, these cells activate MAPK signaling pathways (p38 and JNK), resulting in the secretion of IL-6 and TGF-β secretion. This in turn polarizes naïve CD4+ T cells towards the Th17 phenotype [70]. Furthermore, it has been demonstrated that CD8+ T cells stimulate the Na-Cl co-transporter (NCC) in the distal convoluted tubule (DCT), leading to increased sodium retention and the development of salt-sensitive hypertension [71].

In parallel, aldosterone-induced expression of the small leucine-rich proteoglycan biglycan, released under conditions of cellular stress, serves as a potent DAMP by engaging Toll-like receptors (TLR2 and TLR4) on resident and infiltrating cells, thereby amplifying inflammatory signals via MyD88-dependent NF-κB activation and promoting recruitment of macrophages and Th1/Th17 lymphocytes, implicated in vascular remodeling and cardiac fibrosis [38,72,73]. The transcriptional activity of the MR is finely modulated by co-regulators such as members of the SRC family and p300/CBP and is further potentiated by interactions with the glucocorticoid receptor (GR), adding another layer of tissue-specific regulation to aldosterone signaling [74,75]. Moreover, microRNAs such as miR-181a, miR-192, miR-802, and others regulate key elements of the RAAS and ion channel expression, with altered miRNA expression profiles implicated in the development of hypertension, heart failure, and kidney disease [76,77].

Growing evidence suggests that biological sex influences aldosterone biosynthesis and MR activation. Estrogens enhance aldosterone production via upregulation of angiotensin II receptors and CYP11B2 expression in the zona glomerulosa [78]. Moreover, estrogen signaling via GPER modulates downstream MR signaling, potentially exerting protective vascular effects in premenopausal women [59]. After menopause, the decline in estrogen correlates with increased aldosterone activity, vascular stiffness, and salt sensitivity, potentially contributing to the higher prevalence of resistant hypertension and HFpEF in older women [79]. These sex-related differences may also extend to differential responses to MR antagonists, with some data suggesting greater reductions in albuminuria and blood pressure in women, though evidence remains limited [80].

### 2.4. The Aldosterone-Sensitive Distal Nephron

The primary physiological stage for aldosterone’s renal actions is the aldosterone-sensitive distal nephron (ASDN). This collective term refers to the terminal segments of the nephron, comprising the late portion of the distal convoluted tubule (specifically the DCT2 segment), the connecting tubule (CNT), and the entire collecting duct (CD) system [81]. Although this segment accounts for only about 5–10% of total filtered sodium reabsorption, its activity is the ultimate determinant of sodium balance, extracellular fluid volume, and potassium homeostasis [82]. The ASDN is not a uniform structure but a heterogeneous mosaic of distinct cell types, each with specialized transport functions, allowing for the differential regulation of various physiological tasks under the control of a single hormone. The key cellular actors on this stage and how they are directed by aldosterone are shown in Table 1. The differential distribution of transporters along the ASDN, with the NCC being predominant in the DCT and ENaC/ROMK dominating in the CNT and CD, allows for context-dependent regulation, to dissociate sodium retention from potassium secretion [83].

**Table 1:** Cellular and molecular basis of aldosterone action in the distal nephron.

Cell Type and Location	Transporters	Function	Regulation by Aldosterone	Net Physiological Consequence
DCT Cell	Apical: • Na^+^-Cl^−^ Cotransporter (NCC; *SLC12A3*) Basolateral: • Na^+^/K^+^-ATPase • Cl^−^ Channel (ClC-Kb)	Electroneutral Na^+^ and Cl^−^ reabsorption.	• Ang II strongly activates NCC via the WNK-SPAK/OSR1 kinase pathway independent of aldosterone [84]. • Aldosterone can increase total NCC protein abundance [85] and does not require Ang II to do so via the WNK4-SPAK pathway [86]. • NCC activity is inhibited by high dietary K^+^ [87].	Major site of action for thiazide diuretics. Critical for dissociating Na^+^ retention from K^+^ secretion.
Principal Cell Connecting Tubule (CNT), Cortical Collecting Duct (CCD)	Apical: • Epithelial Na^+^ Channel (ENaC) • Renal Outer Medullary K^+^ Channel (ROMK; *KCNJ1*) • Big K^+^ (BK) Channel (Flow-dependent) Basolateral: • Na^+^/K^+^-ATPase	Na^+^ reabsorption K^+^ secretion	• Aldosterone increases transcription, translation, and membrane insertion/activity of ENaC subunits and the Na^+^/K^+^-ATPase [45]. • Aldosterone upregulates the serum- and glucocorticoid-induced kinase 1 (SGK1), which phosphorylates and inactivates the E3 ubiquitin ligase Nedd4-2, preventing ENaC degradation [45,88]. • Aldosterone promotes ROMK and BK channel activity for K^+^ secretion [45].	Na^+^ retention and ECF volume expansion K^+^ excretion (kaliuresis) Contributes to hypertension in states of aldosterone excess.
Type A (α) Intercalated Cell CNT, CCD, Outer Medullary Collecting Duct (OMCD)	Apical: • Vacuolar H^+^-ATPase (V-ATPase) • H^+^/K^+^-ATPase (HKA) Basolateral: • Cl^−^/HCO_3_^−^ Exchanger 1 (AE1; SLC4A1)	H^+^ secretion HCO_3_^−^ reabsorption K^+^ reabsorption (via HKA)	• Aldosterone increases activity and membrane density of the apical V-ATPase [89,90,91]. • Aldosterone upregulates HKA activity, particularly during hypokalemia [92,93].	Systemic acid excretion and urine acidification Contributes to metabolic alkalosis and hypokalemia in states of aldosterone excess.
Type B (β) Intercalated Cell, CNT, CCD	Apical: • Pendrin (Cl^−^/HCO_3_^−^ Exchanger; SLC26A4) • Na^+^-driven Cl^−^/HCO_3_^−^ exchanger (SLC4A8) Basolateral: • Vacuolar H^+^-ATPase (V-ATPase)	HCO_3_^−^ secretion Cl^−^ reabsorption H^+^ reabsorption	• Aldosterone (especially when co-signaled by low ang II) suppresses pendrin activity [94,95].	Systemic base excretion and urine alkalinization. Correction of metabolic alkalosis. Aldosterone-mediated inhibition promotes Cl^−^-sparing Na^+^ retention.
Non-A, Non-B Intercalated Cell, CNT, CCD	Apical: • Expresses both V-ATPase and Pendrin.	A hybrid phenotype capable of either H^+^ or HCO_3_^−^ secretion.	Regulation is complex and less defined; thought to contribute to the fine-tuning of acid–base balance by adapting its function [4].	Provides plasticity to the renal response to acid–base disturbances.

Abbreviations: DCT, distal convoluted tubule; CNT, connecting tubule; CCD, cortical collecting duct; OMCD, outer medullary collecting duct; ENaC, epithelial sodium channel; ROMK, renal outer medullary potassium channel; BK, big potassium channel; NCC, Na^+^-Cl^−^ cotransporter; SGK1, serum- and glucocorticoid-induced kinase 1; Na^+^/K^+^-ATPase, sodium–potassium ATPase; V-ATPase, vacuolar H^+^-ATPase; HKA, H^+^/K^+^-ATPase; AE1, anion exchanger 1; ClC-Kb, chloride channel Kb; SLC, solute carrier family; Ang II, angiotensin II; ECF, extracellular fluid.

**Figure 4:**
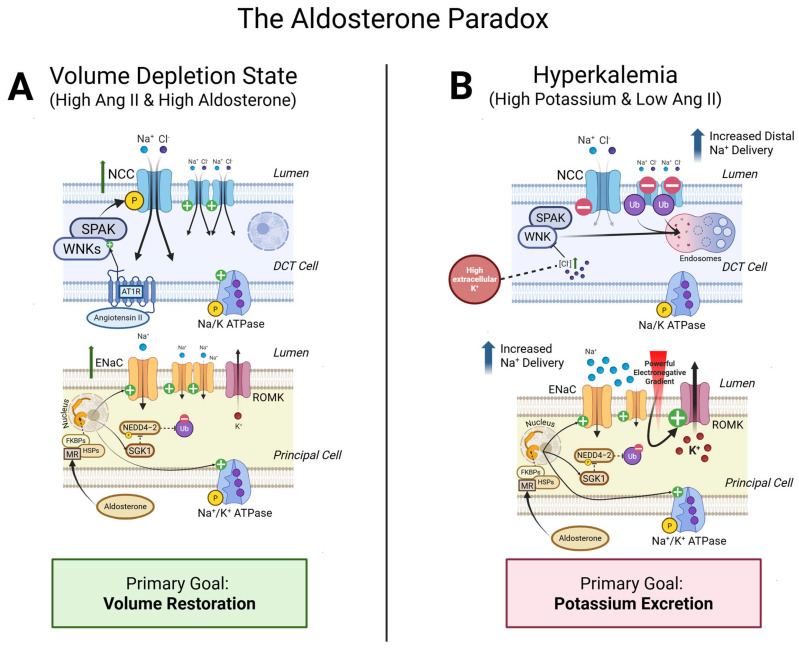
**The aldosterone paradox.** Aldosterone’s physiological effect is determined by co-signals, allowing the kidney to dissociate sodium (Na^+^) retention from potassium (K^+^) secretion. (**A**) In volume depletion, high Angiotensin II activates the Na-Cl cotransporter (NCC) via the WNK-SPAK pathway to maximize Na^+^ reabsorption and restore volume. (**B**) In hyperkalemia, high plasma K^+^ inhibits NCC, which increases Na^+^ delivery to the collecting duct. This allows aldosterone’s stimulation of ENaC to generate a strong electrochemical gradient that drives robust K^+^ secretion.

In states of volume depletion (high Ang II, high aldosterone), the primary goal is to conserve sodium and restore volume. Ang II acts as a powerful stimulator of the NCC in the DCT [84]. In concert, aldosterone stimulates both NCC and ENaC [45,85,86]. In states of hyperkalemia (high K^+^, high aldosterone, low Ang II), the primary goal is to excrete excess potassium. Critically, high plasma K^+^ (and the accompanying low Ang II) inhibits the activity of NCC in the DCT [87]. This prevents premature sodium reabsorption, thereby ensuring robust delivery of sodium and fluid downstream. Aldosterone’s stimulation of ENaC and ROMK can now proceed with maximal efficiency [45]. The ample luminal sodium provides the necessary substrate for ENaC-mediated reabsorption, which in turn generates the strong lumen-negative potential required to drive vigorous potassium secretion through ROMK [96]. This paradoxical regulation (aldosterone paradox) demonstrates that aldosterone does not act in a vacuum. It is a DCT orchestrator that works in concert with co-signals (Ang II and K^+^) and modulates the responsiveness of different effectors (NCC vs. ENaC/ROMK) in different parts of the nephron [97]. This creates a highly sophisticated, context-dependent response that is far more nuanced than a simple, monolithic command to retain sodium and excrete potassium (Figure 4).

## 3. Clinical Syndromes

Disorders of mineralocorticoid receptor (MR) overactivation can be classified into three key groups based on the underlying levels of aldosterone and renin. Primary hyperaldosteronism is characterized by the autonomous production of aldosterone by the adrenal glands, leading to high aldosterone, suppressed plasma renin activity (PRA), and hypertension [98,99,100,101]. In contrast, secondary hyperaldosteronism is driven by the overactivation of the RAAS, resulting in both high PRA and high aldosterone [102]. A third, crucial category is pseudo-hyperaldosteronism, which clinically mimics primary hyperaldosteronism, presenting with hypertension and hypokalemia, but is defined by suppressed PRA and suppressed aldosterone [97]. Pseudo-hyperaldosteronism includes MR-dependent forms (apparent mineralocorticoid excess, licorice ingestion, severe forms of Cushing’s Syndrome) and the MR-independent Liddle syndrome (ENaC gain-of-function), which is unresponsive to mineralocorticoid receptor antagonists (MRAs) but corrected by ENaC blockade (Table 2). To provide a coherent framework for this differential diagnosis, Table 2 offers a classification of these disorders, summarizing their core pathophysiology and key biochemical signatures. A more exhaustive list including syndromes of aldosterone deficiency and resistance is provided in Supplemental Table S1.

**Table 2:** Classification of hyperaldosteronism-related clinical disorders.

Category	Condition or Syndrome	Core Pathophysiology	Plasma Aldosterone	Plasma Renin Activity	Aldosterone/Renin Ratio	Serum K^+^	Blood Pressure
Primary hyperaldosteronism [98]	Conn’s Syndrome [103] Bilateral Adrenal Hyperplasia [104]	Autonomous aldosterone production	High	Suppressed/ Low	High	Low/ Normal	High
	Familial Hyperaldosteronism (FH I-IV) [105,106]	Genetic variants (*CYP11B1/CYP11B2*, *CLCN2*, *KCNJ5*, *CACNA1H*) causing autonomous aldosterone production	High	Suppressed/ Low	High	Low/ Normal	High
Secondary hyperaldosteronism [102]	Renovascular Hypertension [107] Reninoma [108]	Renin overproduction (physiological or neoplastic)	High	High	Low/ Normal	Low/ Normal	High
	Heart Failure [109] Cirrhosis [110,111] Nephrotic Syndrome [111]	RAAS activation due to low effective circulating volume	High	High	Low/ Normal	Normal/ High	Low/ Normal
	Salt-Wasting Tubulopathies [112,113]	RAAS activation from renal salt wasting	High	High	Low/ Normal	Low	Low/ Normal
Pseudo hyperaldosteronism [114]	Liddle Syndrome [115]	Gain-of-function variants in ENaC (*SCNN1A*, *SCNN1B*, *SCNN1C*) [116]	Suppressed/ Low	Suppressed/ Low	Low/Variable	Low	High
	Apparent Mineralocorticoid Excess (AME) [117] Licorice [118]	Impaired cortisol inactivation by 11β-HSD2	Suppressed/ Low	Suppressed/ Low	Low/Variable	Low	High
	Cushing’s Syndrome (severe) [119]	MR activation by cortisol excess	Suppressed/ Low	Suppressed/ Low	Low/Variable	Low	High
	Exogenous Mineralocorticoids (Fludrocortisone) [120]	Exogenous MR agonist	Suppressed/ Low	Suppressed/ Low	Low/Variable	Low	High

This table stratifies the key clinical syndromes that present as mineralocorticoid excess. The classification is based on the primary pathophysiological driver and the resulting biochemical signature of plasma renin and aldosterone, which is crucial for accurate diagnosis. These conditions are characterized by mineralocorticoid receptor (MR) overactivation, whether through high aldosterone levels (primary and secondary hyperaldosteronism) or aldosterone-independent mechanisms (pseudohyperaldosteronism). For a comprehensive classification of syndromes related to aldosterone deficiency or resistance, please see Supplemental Table S1. Abbreviations: FH, Familial Hyperaldosteronism; MR, Mineralocorticoid Receptor; RAAS, Renin–Angiotensin–Aldosterone System.

While the clinical syndromes of aldosterone dysregulation are diverse, the consequences of MR overactivation in the setting of CKD represent a particularly critical and evolving area. In patients with CKD, traditional risk factors for progression and cardiovascular mortality are amplified by non-classical MR activation, which drives inflammation, fibrosis, and oxidative stress within the kidneys and vasculature [121,122,123]. This MR-driven pathology persists even in patients with aldosterone levels within the normal range and contributes significantly to the residual cardiorenal risk that is not mitigated by standard-of-care therapies like renin–angiotensin system inhibitors [124]. Therefore, understanding the rationale for and application of therapies that block this pathway has become a cornerstone of modern nephrology. The following sections will focus specifically on the treatment of hyperaldosteronism and MR overactivation in the context of CKD, reviewing the evidence for established MRAs and exploring the next generation of agents designed to offer more targeted and safer intervention.

## 4. Treatment of Hyperaldosteronism in Chronic Kidney Disease

### 4.1. Molecular Rationale for Mineralocorticoid Receptor Antagonists

Recent research has elucidated MR’s central role in driving inflammation, fibrosis, and oxidative stress, fundamental processes in CKD pathophysiology and its cardiovascular complications [34,125]. MR expression extends significantly beyond epithelial cells, including podocytes [34,126,127], mesangial cells [34], fibroblasts [128], endothelial cells [129], cardiomyocytes [33], and immune cells, such as macrophages and T lymphocytes [34,128]. Activation of MR in these cells initiates deleterious pathways independently from systemic blood pressure modulation [34]. Moreover, despite effective initial suppression of the RAAS, long-term treatment with ACE inhibitors or ARBs frequently results in a phenomenon known as aldosterone breakthrough, a gradual and paradoxical rise in aldosterone levels occurring in up to 50% of patients [130]. Aldosterone breakthrough is now recognized as a central mechanism of residual cardiorenal risk and strongly supports the use of adjunctive therapies. Furthermore, a key therapeutic benefit of MRAs is their antiproteinuric effect, derived by the mitigation of MR-driven inflammation and fibrosis in podocytes and mesangial cells, thus improving glomerular selectivity, independently of blood pressure reduction [121,131,132,133]. Proteinuria, and specifically albuminuria, is not only a marker of kidney damage but also an independent and modifiable risk factor for the progression of CKD and cardiovascular events [134,135].

Clinically, steroidal MRAs, spironolactone and eplerenone, initially demonstrated marked cardiovascular benefits in heart failure (RALES, EPHESUS, EMPHASIS-HF trials) [136,137,138], but their applicability in CKD is limited due to significant risks of hyperkalemia, endocrine side effects, and unclear benefits on CKD progression [139,140]. The negative results from the BARACK-D trial further highlighted the limitations of steroidal MRAs in moderate CKD populations [141]. Finerenone, a novel non-steroidal MRA, has substantially reshaped this therapeutic landscape. Its distinct chemical structure confers high MR selectivity without hormonal side effects, exhibiting balanced cardiac and renal tissue distribution [123]. Landmark trials, FIDELIO-DKD and FIGARO-DKD, demonstrated significant reductions in both kidney and cardiovascular outcomes in diabetic kidney disease (DKD), with significant reduction in the urinary albumin-to-creatinine ratio, independent of major blood pressure changes, underscoring finerenone’s direct anti-inflammatory and anti-fibrotic actions [142,143,144]. These advances highlight critical pharmacologic and mechanistic distinctions between steroidal and non-steroidal MRAs, summarized in Table 3. Safety profiles from these trials revealed a manageable hyperkalemia risk, significantly lower compared to steroidal MRAs, further enhanced by meticulous potassium monitoring and management protocols [145]. This favorable balance positions finerenone as a cornerstone in CKD management, complementing existing therapies such as RAS inhibitors and SGLT2 inhibitors [146]. Emerging non-steroidal MRAs, including esaxerenone and apararenone, have shown promise in specific patient populations, reducing albuminuria particularly within hypertensive and diabetic cohorts, yet require broader validation through global trials [147,148]. Conversely, ocedurenone, targeting resistant hypertension in advanced CKD, failed to demonstrate clinical efficacy in the pivotal CLARION-CKD trial, underscoring complexities in developing therapies for advanced CKD stages. Guidelines now advocate integrating non-steroidal MRAs in multi-targeted therapeutic strategies for CKD in diabetic patients, reflecting a paradigm shift towards addressing MR-mediated inflammation and fibrosis as central therapeutic goals [149].

**Table 3:** Comparative overview of steroidal and non-steroidal MRAs in the context of CKD.

Feature	Steroidal MRAs (Spironolactone, Eplerenone)	Non-Steroidal MRA (Finerenone)	Emerging Non-Steroidal MRAs (Esaxerenone, Apararenone)
Structure	Steroidal	Non-steroidal (Dihydropyridine-based)	Non-steroidal
MR Selectivity	Spironolactone: Low Eplerenone: Moderate–High	High	Generally High
Androgen/Progesterone Receptor Binding	Spironolactone: Significant Eplerenone: Minimal	Minimal/Negligible	Minimal/Negligible
Mechanism at MR	Competitive Antagonist (Potentially Partial Agonist)	Competitive Antagonist/Inverse Agonist (Blocks pathological co-factor recruitment)	Competitive Antagonist (mechanisms may vary)
Potency (vs. Spironolactone)	Eplerenone: Lower	Similar/Higher	Variable
Metabolites	Spironolactone: Active, long half-life Eplerenone: Inactive	Inactive, short half-life	Generally inactive, half-lives vary
Tissue Distribution	Preferential Kidney Accumulation	Balanced Heart/Kidney Distribution	Variable/Under investigation
BP Lowering Effect	Spironolactone: effective, cornerstone therapy for resistant hypertension [150] Eplerenone: modest [136,138]	Modest [151]	Significant [150,152]
Proven CKD Progression Benefit (Hard Outcomes)	No (BARACK-D negative) [139]	Yes (FIDELIO-DKD, FIDELITY)	Limited/Under investigation
Proven CV Benefit in CKD	No (BARACK-D negative [139]), Yes (in HFrEF [140])	Yes (FIGARO-DKD, FIDELITY)	Limited/Under investigation
Albuminuria Reduction	Yes [152]	Yes (proven in trials)	Yes (Esaxerenone [147], Apararenone [148])
Hyperkalemia Risk in CKD	High, often dose-limiting [140]	Moderate, manageable with monitoring (lower relative risk vs. steroidal likely)	Appears lower than steroidal, requires confirmation in large trials
Hormonal Side Effects	Spironolactone: Common [153] Eplerenone: Rare [153]	Rare/Absent	Rare/Absent
Primary Indication in CKD Context	HFrEF [154], Resistant HTN (with caution)	Cardiorenal risk reduction in T2D with albuminuric CKD (ACR > 30 mg/g)	Hypertension/DKD (Esaxerenone, Japan); DKD (Apararenone, Japan); Uncontrolled HTN (Ocedurenone, failed Phase III)

Abbreviations: CKD: chronic kidney disease, DKD: diabetic kidney disease, HFrEF: heart failure reduced ejection fraction, HTN: hypertension, T2D: type 2 diabetes.

### 4.2. Selective Aldosterone Synthase Inhibitors

Selective aldosterone synthase inhibitors (ASIs) represent a class of agents that suppress aldosterone production by inhibiting CYP11B2 [151]. By targeting the source of aldosterone rather than its receptor, ASIs offer the potential to mitigate both genomic and non-genomic effects of the hormone [155]. A central challenge in ASI development has been achieving sufficient selectivity over CYP11B1, the structurally similar enzyme responsible for cortisol synthesis [28]. Osilodrostat (LCI699), a potent dual inhibitor of CYP11B1 and CYP11B2, confirmed its ability to lower aldosterone levels and correct hypokalemia in patients with primary aldosteronism [156], but had clinically meaningful cortisol suppression [157]. Its modest selectivity ratio (CYP11B2:CYP11B1 ≈ 6–10:1) [158] led to side effects such as adrenal insufficiency, androgen excess, and QTc prolongation [159], and ultimately to its repurposing for Cushing’s disease [157]. In contrast, baxdrostat is a next-generation ASI engineered for high selectivity (CYP11B2:CYP11B1 > 100:1) [158]. Preclinical and early-phase clinical data demonstrate that baxdrostat significantly suppresses plasma aldosterone without affecting cortisol synthesis, even under ACTH stimulation [160,161]. Preliminary post hoc data from BrigHTN suggest baxdrostat may reduce urinary albumin-to-creatinine ratio (UACR) [162]. Its role in CKD is under evaluation (NCT06268873, NCT06742723). Lorundrostat, another highly selective, next-generation ASI, has been shown to significantly reduce blood pressure in patients with uncontrolled or resistant hypertension, in a dose-dependent manner [163,164]. Based on these results, the ongoing Explore-CKD Phase 2 trial (NCT06150924) has been designed to evaluate the efficacy of lorundrostat in patients with CKD and significant albuminuria (uACR 200–5000 mg/g).

### 4.3. Future Therapeutic Frontiers

Future therapeutic strategies in aldosterone-related diseases are increasingly focusing beyond classical genomic MR-mediated pathways [153,165]. Rapid, non-genomic actions of aldosterone are frequently insensitive to MRAs and are instead orchestrated by a subset of membrane-associated receptors and scaffolding proteins [60]. Emerging therapeutic strategies are now targeting these non-classical pathways to overcome the limitations of current MRAs and ASIs. Table 4 enlists emerging molecular targets. One promising avenue involves the modulation of GPER activity, either by using selective agonists to harness beneficial, estrogen-like effects or by employing antagonists (G15, G36) to specifically block aldosterone-mediated detrimental signaling [59,60]. Parallel strategies aim to mitigate inflammation and fibrosis through direct inhibition of the NLRP3 inflammasome using agents such as MCC950 and the orally active compound dapansutrile (OLT1177), both of which have shown efficacy in reducing caspase-1 activity, IL-1β/IL-18 production, and subsequent tissue injury in preclinical models of salt-sensitive hypertension and cardiac dysfunction [64,66,166,167]. A Phase IB trial in patients with stable systolic heart failure found dapansutrile to be safe and well-tolerated, with the highest dose showing preliminary efficacy signals, including a significant improvement in left ventricular ejection fraction and exercise time [168]. Its broad clinical pipeline, with trials ranging from acute gout (Phase III, NCT05658575) to type 2 diabetes (Phase II, NCT06047262) and post-myocardial infarction imaging (NCT05880355), underscores the promise of targeting the NLRP3 inflammasome for a range of chronic diseases.

Additional avenues include the blockade of TLR signaling pathways, with molecules like resatorvid (TAK-242) targeting TLR4, to attenuate biglycan-triggered inflammatory cascades [169,170,171], and strategies that focus on neutralizing soluble biglycan directly, thereby preventing its interaction with TLRs and subsequent amplification of immune responses [72,169]. Resatorvid was advanced all the way to a large-scale Phase III clinical trial for the treatment of severe sepsis, but failed to meet its primary endpoint, effectively halting its progress as a viable therapeutic candidate [172].

Looking toward future clinical guidelines, pathways with well-defined downstream effects and available investigational agents are the most likely to become recommended interventions. Given the robust preclinical data, targeting the NLRP3 inflammasome appears to be one of the most promising future strategies to address residual inflammatory risk.

At the same time, the advent of miRNA-based therapeutics, whether by administration of miRNA mimics to restore downregulated protective miRNAs or by employing antagomirs to suppress deleterious miRNAs, offers the potential to correct dysregulated pathways at the post-transcriptional level, thereby fine-tuning the RAAS and reducing aldosterone-mediated toxicity [77]. Furthermore, immunomodulatory approaches that seek to re-balance the immune cell milieu, such as strategies to enhance regulatory T cell (Treg) function or to inhibit pathogenic Th17 differentiation, have shown promise in preclinical studies aimed at reducing aldosterone-induced vascular remodeling and fibrosis [64,173,174]. Integral to the successful application of these novel interventions is the development of robust biomarkers that move beyond conventional aldosterone and renin measurements. Biomarkers reflecting pathway-specific activation, such as circulating levels of IL-1β, IL-18, and apoptosis-associated speck-like protein (ASC) specks as surrogates for inflammasome activity, as well as soluble biglycan levels indicating MR-mediated extracellular matrix remodeling and TLR engagement, offer promising avenues for patient stratification and therapeutic monitoring [64,65,73]. Profiling circulating microRNA signatures along with quantification of immune cell ratios could provide a composite biomarker panel that guides precision medicine approaches and informs the selection of appropriate targeted therapies [174,175,176]. In clinical practice, identifying which patients harbor subclinical MR overactivation, despite ‘normal’ aldosterone levels, remains a challenge. Biomarkers capturing tissue-level activity rather than circulating hormone levels may ultimately redefine therapeutic indications. Yet, most of these candidates remain investigational. Their specificity, reproducibility, and predictive utility must be validated in prospective, longitudinal cohorts. Furthermore, integration into clinical workflows demands the development of robust, scalable assays and consensus on threshold values. Looking forward, the integration of advanced experimental models, such as three-dimensional organoids, microfluidic “organ-on-a-chip” systems, and genetically modified animal models with cell-specific deletions, combined with comprehensive multi-omics analyses, is expected to accelerate the validation of these biomarkers and facilitate the translation of preclinical findings into clinical practice [177].

**Table 4:** Summary of emerging molecular targets beyond MRAs/ASIs.

Target/Pathway	Key Molecular Components	Primary Mechanism(s)	Implicated Pathologies
Non-Genomic Signaling (Membrane MR/Scaffolds) [178]	MR, Striatin, Cav-1, Kinases (ERK, PKC), EGFR	Rapid kinase activation (ERK, PKC), Ion flux modulation, EGFR transactivation	CV remodeling, Renal transport regulation
GPER Signaling [7]	GPER (GPR30), Gs, GRKs, ERK, PI3K	Aldosterone binding, Rapid kinase activation (ERK), Potential cAMP/PI3K modulation	Hypertension, Vascular dysfunction, Inflammation, Apoptosis
Inflammation/Fibrosis [179] (General)	Immune cells (Macs, T cells), Cytokines (TNFα, IL-6, IL-1β), Chemokines, Adhesion molecules, ROS, Fibroblasts, ECM	Immune cell activation/infiltration, Cytokine release, Oxidative stress, Fibroblast proliferation, ECM deposition	CV inflammation/fibrosis, Renal inflammation/fibrosis
NLRP3 Inflammasome [64]	NLRP3, ASC, Caspase-1, IL-1β, IL-18	Aldosterone-induced ROS/NF-κB activation, Inflammasome assembly, Cytokine maturation	Vascular dysfunction/remodeling, Podocyte injury, Renal inflammation
Biglycan/TLR Signaling [72]	Biglycan, TLR4, TLR2, MyD88, TRIF, NF-κB, Chemokines (CCL3, CXCL9/10, CCL20)	MR-induced Biglycan upregulation, TLR activation by soluble Biglycan, Immune cell recruitment (Macs, Th1/Th17)	Glomerular injury, Renal inflammation/fibrosis
MR Co-regulators/GR Interaction [74]	SRCs, p300/CBP, GR	Modulation of MR transcription initiation/efficiency, GR potentiation of MR activity	Tissue-specific aldosterone sensitivity
MicroRNAs [76]	miR-192, miR-802, miR-194, miR-181a, miR-663, miR-483 etc.	Post-transcriptional regulation of RAAS components and effectors (mRNA degradation/translation inhibition)	Hypertension, Kidney disease, Electrolyte balance

Abbreviations: MR, mineralocorticoid receptor; MRAs, mineralocorticoid receptor antagonists; ASIs, aldosterone synthase inhibitors; GPER, G protein–coupled estrogen receptor; ERK, extracellular signal–regulated kinase; PKC, protein kinase C; EGFR, epidermal growth factor receptor; PI3K, phosphoinositide 3-kinase; ROS, reactive oxygen species; NLRP3, NOD-, LRR- and pyrin domain–containing protein 3; ASC, apoptosis-associated speck-like protein containing a CARD; IL-1β, interleukin 1 beta; IL-18, interleukin 18; TLR, Toll-like receptor; MyD88, myeloid differentiation primary response 88; TRIF, TIR-domain-containing adapter-inducing interferon-β; NF-κB, nuclear factor kappa B; SRC, steroid receptor coactivator; CBP/p300, CREB-binding protein/E1A-binding protein p300; GR, glucocorticoid receptor; ECM, extracellular matrix; Th1/Th17, T helper type 1/type 17 cells.

## 5. Conclusions

Once an adaptive triumph of terrestrial physiology, aldosterone has become, in the modern era, a double-edged hormone, indispensable for homeostasis, yet deeply implicated in inflammation, fibrosis, and cardiorenal injury. Its action extends far beyond epithelial ion transport, orchestrating complex metabolic responses through both classical and non-classical signaling. While mineralocorticoid receptor antagonists and aldosterone synthase inhibitors have opened new therapeutic avenues, their clinical potential remains partially untapped, hindered by an outdated reliance on circulating hormone levels and a one-size-fits-all treatment paradigm. We argue that the future of aldosterone research lies not in blocking its presence, but in decoding its logic, disentangling context-dependent actions, identifying tissue-specific vulnerabilities, and leveraging pathway-informed biomarkers to guide therapy. In this light, aldosterone should no longer be viewed merely as a hormone to suppress, but as a modular signal to be reprogrammed. Reframing aldosterone from endocrine villain to therapeutic interface may be the key to resolving residual risk in cardiorenal disease and ushering in a new era of precision nephrology.

## Supplementary Materials

The following supporting information can be downloaded at: https://www.mdpi.com/article/10.3390/ijms26188829/s1 [180,181,182,183,184,185,186,187,188].
