# Supplementary material for: Aldosterone: From Essential Tubular Regulator to Pathological Driver—Physiology, Disease, and Therapeutic Advances
Source: Int J Mol Sci. 2025 Sep 10;26(18):8829. doi: 10.3390/ijms26188829 (PMC12469253; doi:10.3390/ijms26188829)
Supplement: Supplementary file 1 [file ijms-26-08829-s001.zip › ijms-3808141-supplementary.pdf]

Category	Condition or Syndrome	Core Pathophysiology	Plasma Aldosterone	Plasma Renin Activity	Aldosterone/Renin Ratio	Serum K+	Blood Pressure
Primary hyperaldosteronism [98]	Conn's Syndrome [103] Bilateral Adrenal Hyperplasia [104]	Autonomous aldosterone production	High	Suppressed / Low	High	Low/ Normal	High
	Familial Hyperaldosteronism (FH I-IV) [105,106]	Genetic variants (*CYP11B1*/*CYP11B2*, *CLCN2*, *KCNJ5*, *CACNA1H*) causing autonomous aldosterone production	High	Suppressed / Low	High	Low/ Normal	High
Secondary hyperaldosteronism [102]	Renovascular Hypertension [107] Reninoma [108]	Renin overproduction (physiological or neoplastic)	High	High	Low/ Normal	Low/ Normal	High
	Heart Failure [109] Cirrhosis [110,111] Nephrotic Syndrome [111]	RAAS activation due to low effective circulating volume	High	High	Low/ Normal	Normal/ High	Low/ Normal
	Salt-Wasting Tubulopathies [112,113]	RAAS activation from renal salt wasting	High	High	Low/ Normal	Low	Low/ Normal
Pseudo hyperaldosteronism [114]	Liddle Syndrome [115]	Gain-of-function variants in ENaC (*SCNN1A*, *SCNN1B*, *SCNN1C*) [116]	Suppressed/ Low	Suppressed / Low	Low/Variable	Low	High
	Apparent Mineralocorticoid Excess (AME) [117] Licorice [118]	Impaired cortisol inactivation by 11β-HSD2	Suppressed/ Low	Suppressed / Low	Low/Variable	Low	High
	Cushing's Syndrome (severe) [119]	MR activation by cortisol excess	Suppressed/ Low	Suppressed / Low	Low/Variable	Low	High
	Exogenous Mineralocorticoids (Fludrocortisone) [120]	Exogenous MR agonist	Suppressed/ Low	Suppressed / Low	Low/Variable	Low	High
Primary hypoaldosteronism[180]	Primary Adrenal Insufficiency (Addison's Disease) [181]	Adrenal failure with deficient aldosterone synthesis	Low	High	Low	High	Low
	Aldosterone Synthase Deficiency[182]	Enzymatic defect in aldosterone synthase due to variants in CYP11B2 gene	Low	High	Low	High	Low/ Normal
	Heparin-Induced Hypoaldosteronism[183]	Drug-induced adrenal damage (zona glomerulosa)	Low	High	Low	High	Normal

Secondary hypoaldosteronism[180]	Type 4 Renal Tubular Acidosis	Aldosterone deficiency driven by renal renin insufficiency (due to juxta-glomerulus apparatus damage or functional suppression) due to Diabetic Kidney Disease[184], Chronic Kidney Disease, Autonomic neuropathy, Sickle cell disease, HIV disease or drugs (NSAIDs, COX-2 inhibitors, β-blockers, Calcineurin Inhibitors)	Low	Low	Variable	High	Normal/ High
Aldosterone Resistance States (pseudohypoaldosteronism)[180]	Type 1 (PHA1)[185,186]	Loss-of-function variants in MR or ENaC encoding genes (AD: NR3C2 encoding MR, AR: SCNN1A, SCNN1B, SCNN1G encoding ENaC).	High	High	Low/ Normal	High	Low
	Type 2 (PHA2) or Gordon Syndrome or Familial Hyperkalemic Hypertension[187]	Gain-of-function variants in genes involved with NCC activity (WNK1, WNK4, CUL3, KLHL3)	Normal/ High	Suppressed/ Low	High	High	High
	Transient, due to UTIs, CAKUT or obstructive uropathy[188]	Transient aldosterone resistance induced by renal inflammation/obstruction	High	High	Low/ Normal	High	Low/ Normal

This table provides a comprehensive classification of the full spectrum of aldosterone-related clinical disorders. The disorders are stratified based on the primary mechanism, distinguishing between states of excessive, insufficient, or paradoxical mineralocorticoid receptor (MR) activation. For each condition, the core pathophysiology is described along with the expected biochemical phenotype and clinical presentation. This table is intended as a broad clinical and diagnostic reference. *Abbreviations*: AD, Autosomal Dominant; AME, Apparent Mineralocorticoid Excess; AR, Autosomal Recessive; ARR, Aldosterone/Renin Ratio; BAH, Bilateral Adrenal Hyperplasia; CAKUT, Congenital Anomalies of the Kidney and Urinary Tract; CKD, Chronic Kidney Disease; ENaC, Epithelial Sodium Channel; FH, Familial Hyperaldosteronism; HIV, Human Immunodeficiency Virus; MR, Mineralocorticoid

Receptor; NCC, Na-Cl Cotransporter; NSAIDs, Non-Steroidal Anti-Inflammatory Drugs; PHA, Pseudohypoaldosteronism; PRA, Plasma Renin Activity; RTA, Renal Tubular Acidosis; UTI, Urinary Tract Infection; WNK, With-No-Lysine (Kinase).
